# Multiple imputation for systematically missing effect modifiers in individual participant data meta-analysis

**DOI:** 10.1177/09622802251348800

**Published:** 2025-06-20

**Authors:** Robert Thiesmeier, Scott M Hofer, Nicola Orsini

**Affiliations:** 1 Department of Global Public Health, 27106 Karolinska Institutet, Sweden; 2 Aging Research Center, Department of Neurobiology, Care Sciences and Society, 27106 Karolinska Institutet, Sweden; 3 50680 Pacific Health Research and Education Institute, Honolulu, Hawaii, USA

**Keywords:** Individual participant data meta-analysis, systematically missing data, two-stage meta-analysis, conditional quantile imputation, Monte-Carlo simulation, treatment effect modification

## Abstract

Individual participant data (IPD) meta-analysis of randomised trials is a crucial method for detecting and investigating effect modifications in medical research. However, few studies have explored scenarios involving systematically missing data on discrete effect modifiers (EMs) in IPD meta-analyses with a limited number of trials. This simulation study examines the impact of systematic missing values in IPD meta-analysis using a two-stage imputation method. We simulated IPD meta-analyses of randomised trials with multiple studies that had systematically missing data on the EM. A multivariable Weibull survival model was specified to assess beneficial (Hazard Ratio (HR) = 0.8), null (HR = 1.0), and harmful (HR = 1.2) treatment effects for low, medium, and high levels of an EM, respectively. Bias and coverage were evaluated using Monte-Carlo simulations. The absolute bias for common and heterogeneous effect IPD meta-analyses was less than 0.016 and 0.007, respectively, with coverage close to its nominal value across all EM levels. An uncongenial imputation model resulted in larger bias, even when the proportion of studies with systematically missing data on the EM was small. Overall, the proposed two-stage imputation approach provided unbiased estimates with improved precision. The assumptions and limitations of this approach are discussed.

## 1. Introduction

Individual participant data (IPD) meta-analysis of randomised trials is a key method to identify and investigate differential treatment effects (effect modification) in medical research.^1–4^ Single trials may lack the statistical power to detect detailed subgroup differences in treatment effects.^4–6^ For this reason, IPD meta-analyses provide an important opportunity to increase power to detect genuine effect modification.^2,7^ A two-stage IPD approach for estimating treatment effect modification mitigates aggregation bias by first evaluating the effect modification within each individual study and then combining the results in the second stage.^8^ However, missing data in individual studies are common and pose challenges when performing an IPD meta-analysis. While sporadically missing data can be managed within a single study, systematically missing data might lead to the exclusion of entire studies.

Systematically missing data is present when one or more variables are not available in one or more studies.^9–11^ Variables might be systematically missing for different reasons (e.g., different survey instruments or measurement devices, or lack of information) and often pose practical as well as methodological challenges such as the risk of decreasing the ability to evaluate a broader range of effect modifications. Multiple imputation (MI) is a popular method that can be used to retain studies with systematically missing values in IPD meta-analyses.^10,12–14^ In brief, MI ensures that missing data are replaced by their corresponding imputation samples, resulting in M completed datasets where M is the number of imputations. The substantive (outcome) model is then fit in M imputed datasets. Rubin’s rules are applied to pool the M estimates and take into account the variability in the results between the imputed datasets. An important feature of MI is its flexibility, since the imputation model and the substantive model do not have to be the same. However, the two models have to be *congenial*.^15^ In other words, the imputation and substantive model can be derived from a joint distribution by appropriate conditioning. This implies that for the imputation model to be congenial, it must include all variables that are also used in the substantive model (including the outcome and transformations of variables).^16–18^

While some studies have looked at systematically missing covariates including confounders,^9^ few have assessed scenarios on when a key effect modifier (EM) might be missing. Therefore, in this analysis we consider the use and evaluation of a two-stage imputation approach to impute systematically missing values of an EM in an IPD meta-analysis with a small number of trials. Identifying, validating, and successfully analysing treatment effect modifications in clinical practice is challenging. Therefore, it is crucial to consider all available studies in an IPD meta-analysis to avoid losing vital information.^19^ The rationale for using MI in this context is primarily to retain all studies in the analysis, thereby assessing treatment effect heterogeneity across the entire population.

The remainder of this paper is structured as follows. First, we describe the method employed for imputing systematically missing values. Subsequently, we outline the structure of the simulation study, encompassing the data generating mechanism (DGM), analytical model, and performance criteria.^20,21^ The results of the study are then presented, followed by an application of the imputation method to an IPD meta-analysis of 10 randomised controlled trials assessing the efficacy of postoperative radiotherapy (PORT) in patients with completely resected non-small cell lung cancer. We conclude with a discussion of the strengths and limitations of the proposed approach.

## 2. Conditional quantile imputation

In this section, conditional quantile imputation (CQI) is introduced for discrete systematically missing data in IPD meta-analysis. The approach extends that of the standard approach of imputing categorical data in single studies. An overview of CQI for continuous data can be found elsewhere.^22,23^ An explanation of the notations used throughout the paper follows. Let the index i denote the i th individual in each study. Further, let the index j=1,2,…,J denote the j th study included in the IPD meta-analysis. We consider a discrete variable, $z_{ij}$ , with k=1,2,…,K levels, and, a p -dimensional set of predictors of $z_{ij}$ , $w_{ij}=(w_{1ij},w_{2ij},\ldots ,w_{pij})$ . Suppose the vector $w_{ij}$ is completely observed for all $i\in 1,\ldots ,n_{j}$ and all j∈1,…,J , while $z_{ij}$ can have missing values. Let $M_{j}\subset \{1,\ldots ,n_{j}\}$ denote the set of indexes corresponding to the individuals with missing values for $z_{ij}$ . The cardinality of $M_{j}$ divided by the sample size $n_{j}$ denotes the proportion of missing data for the j th study, that is $FM_{j}=|M_{j}|/n_{j}$ , ranging from 0 (no missing) to 1 (all missing, i.e., systematically missing). Systematically missing data in a single study is therefore present if $FM_{j}=1$ . We identify J=A∪B , with $A=j\in J:FM_{j}=1$ as the set of studies with systematically missing data and $B=j\in J:FM_{j}<1$ as the set of studies that can be used to fit the imputation model. The imputation for a discrete systematically missing variable in an IPD meta-analysis can be achieved according to three steps: (1) Specification of the imputation model; (2) prediction of conditional probabilities; (3) imputations of the systematically missing variable.

### 2.1. The imputation model

An imputation model is specified in studies J∈B . Let $\theta_{ikj}$ denote the probability that $z_{ij}$ is equal to level k conditionally on a set of observed variables, the vector of $w_{ij}$ , $\theta_{ikj}|w_{ij}=P(z_{ij}=k|w_{ij})$ with $\sum_{k=1}^{K}\theta_{k}=1$ . A study-specific multinomial logistic regression model is considered for the discrete EM, $z_{ij}$

(1) $$\ln\;\left(\frac{\theta_{ikj}}{\theta_{irj}}\right)=f_{ikj}(w_{ij})$$

by specifying a baseline level r . Estimates of the regression coefficients, ${\hat{\gamma }}_{ikj}$ , and their variances, $\hat{\text{Var}}({\hat{\gamma }}_{ikj})$ , in the linear predictor $f_{ikj}(w_{ij})$ are obtained using the maximum likelihood method separately for each study with complete or partial information.

### 2.2. Prediction of conditional probabilities

For all studies in J∈B , the estimated regression coefficients ${\hat{\gamma }}_{ikj}$ are combined using the inverse variance method. Specifically, a weighted average of the imputation regression coefficients from all studies in J∈B is taken, with the weights equal to the inverse of the variance, assigning greater weight to coefficients with smaller variances.^24^ The combined average regression coefficients are denoted by ${\bar{\gamma }}_{ik}$ . An estimate of the variance of ${\bar{\gamma }}_{ik}$ , denoted by $\hat{\text{Var}}({\bar{\gamma }}_{ik})$ , is estimated using the inverse of the sum of weights

(2) $$\hat{\text{Var}}({\bar{\gamma }}_{ik})={\left(\sum_{J\in B}w_{j}\right)}^{-1}$$

where $w_{j}=\hat{\text{Var}}({\hat{\gamma }}_{ikj}{)}^{-1}$ is the weight assigned to study j . The predicted conditional probabilities of falling into the levels of the systematic missing predictor are denoted by ${\hat{\theta }}_{ik}=\hat{P}(z_{i}=k|w_{i})$ . Next, for each regression coefficient ${\bar{\gamma }}_{ik}$ in studies J∈B , including the intercept, and for all linear predictors in the multinomial logistic regression model, a value is drawn from a normal distribution with the mean equal to the average regression coefficients ${\bar{\gamma }}_{ik}$ and standard deviation equal to the square root of the estimated variance of the pooled coefficients.^25^ In total, for every imputation, there are (p+1)×(K−1) independent draws from univariate normal distributions. Specifically, each draw is given by

(3) $$\gamma_{ik}^{*}∼N\left({\bar{\gamma }}_{ik},\sqrt{\hat{\text{Var}}({\bar{\gamma }}_{ik})}\right)$$

The resulting draws, $\gamma_{ik}^{*}$ , are used to define linear predictors, ${\bar{f}}_{ik}(w_{i})$ , which are then utilised to compute conditional predicted probabilities of the missing EM in studies J∈A , with r as the lowest level

(4) $${\hat{\theta }}_{ik}=\left\{ \begin{array}{ll} \frac{1}{1+\sum_{k=2}^{K}e^{{\bar{\;f}}_{ik}(w_{i})}} & \text{if }k=r\\ \frac{e^{{\bar{\;f}}_{ik}(w_{i})}}{1+\sum_{k=2}^{K}e^{{\bar{\;f}}_{ik}(w_{i})}} & \text{if }k>r \end{array}\right.$$

### 2.3. Imputations

For all studies in J∈A , the inverse of the cumulative distribution function (CDF) – *quantile function* – is used to assign a value to the missing EM, $z_{i}$ , given the vector of observed predictors $w_{i}$ . The conditional predicted CDF, ${\hat{\Theta }}_{ik}$ , is equal to the sum of the probabilities of the EM being less than or equal to k :

(5) $${\hat{\Theta }}_{ik}=\hat{P}(z_{i}\leq k|w_{i})=\sum_{k=1}^{K}{\hat{\theta }}_{ik}$$

An imputation of the missing values of the EM in any study with systematic missing $z_{i}$ is obtained by inverting the CDF of a continuous random uniform distribution U∼Uniform(0,1) . The imputed value of the EM is obtained by mapping such random draw from a uniform distribution to the predicted cumulative probabilities ${\hat{\Theta }}_{ik}$ .

## 3. Simulation

### 3.1. Data generating mechanism

We first describe the DGM for the simulation of a single trial followed by a description of the DGM of multiple trials forming the IPD meta-analysis. We then outline the different scenarios considered for the simulation study.

#### 3.1.1. Single trial

We considered a randomised controlled trial ( n=500 ) with a time-to-death outcome, one binary treatment, and one discrete EM. The following random variables defined a single trial:

-
x is a binary treatment (randomly allocated) generated from a Bernoulli distribution with probability of 0.5.
-
z is a discrete EM, modifying the effect of x on the outcome at different levels of z . The EM, z , was generated by categorising a random uniform distribution over a (0,1) interval into 3 values (1, 2, and 3) using 0.4, 0.8, and 1.0 as cumulative probabilities.
-
t is the time from baseline to death (in years) or end of follow-up (10 years), whichever came first.

The outcome random variable, T , denoting individual time-to-death conditional on the treatment x , covariate z (modelled with 3−1=2 indicator variables) as well as their product terms was described by a multivariable Weibull survival model as follows:

(6) $$\begin{array}{c} P(T>t)=S(t)=e^{-\lambda t^{\gamma }} \end{array}$$

(7) $$\begin{array}{c} t_{i}={\left[-\frac{\ln\;(S_{i})}{\lambda_{i}}\right]}^{1/\gamma } \end{array}$$

(8) $$\begin{array}{c} \lambda_{i}=e^{\beta_{0}+\beta_{1}x_{i}+\beta_{2}I(z_{i}=2)+\beta_{3}I(z_{i}=3)+\beta_{4}x\cdot I(z_{i}=2)+\beta_{5}x_{i}\cdot I(z_{i}=3)} \end{array}$$

The shape parameter was defined as γ=1.4 signifying an increase of the baseline mortality rate over the follow-up period. Drawing a random value from a continuous uniform distribution for the survival probability, S , over a 0 to 1 interval, a random value of the individual time-to-death outcome conditionally on x and z was generated as follows:

(9) $$t_{i}={\left[-\frac{\ln\;(S_{i})}{e^{\beta_{0}+\beta_{1}x_{i}+\beta_{2}I(z_{i}=2)+\beta_{3}I(z_{i}=3)+\beta_{4}x_{i}\cdot I(z_{i}=2)+\beta_{5}x_{i}\cdot I(z_{i}=3)}}\right]}^{1/1.4}$$

Any death beyond the follow-up time was set to 10 years and considered censored (i.e., still alive at the end of the follow-up). An indicator variable d=I(t<10) was created to take into account censoring in the estimation of the parameters of the survival model.

#### 3.1.2. Multiple trials

A common and heterogeneous DGM for the generation of multiple trials was considered. We now describe the parameters underlying the Weibull survival model for a common and heterogeneous treatment effect modification.

**Common treatment effect modification**: The primary characteristic of common treatment effect modification is that the parameter values across the studies do not differ from one another. In other words, all trials in the IPD meta-analysis share a single fixed parameter value for the effect modification. The following values of the parameters were defined:

-
$\beta_{0}$ : The mortality rate when all predictors were set to 0 – the intercept – was 10 deaths per 1000 person-years resulting in the regression coefficient $\ln\;\left(\frac{10}{1000}\right)=-4.605$ .

The hypothesised treatment effect modification underlying the individual time-to-event outcomes was a beneficial $\left(HR_{x|z=1}=0.8\right)$ , null $\left(HR_{x|z=2}=1.0\right)$ , and harmful $\left(HR_{x|z=3}=1.2\right)$ treatment effect for low, medium, and high levels of z , respectively. As such, we set the following parameters:

-
$\beta_{1}$ : The beneficial effect of treatment x when z=0 resulted in the regression coefficient $\beta_{1}=\ln\;(0.80)=-0.223$ .

The EM, z , increased the mortality rate in the control group:

-
$\beta_{2}$ : The log mortality hazard ratio(HR) was set to $\beta_{2}=\ln\;(1.2)=0.182$ for the indicator variable I(z=2) .
-
$\beta_{3}$ : The log mortality HR for the indicator variable I(z=3) was set to $\beta_{3}=\ln\;(1.5)=0.405$ .

Next, the regression coefficients of the two product terms between the treatment, x , and the EM, z , were derived from the following assumed interaction mechanism:

-
$\beta_{4}$ : Given $HR_{x|z=2}=1.0$ , it follows that the regression coefficient of the first product term x⋅I(z=2) was set to $\beta_{4}=\ln\;(1)-\ln\;(0.8)=0.223$ .
-
$\beta_{5}$ : Given $HR_{x|z=3}=1.2$ , we have that the regression coefficient of the second product term x⋅I(z=3) was set to $\beta_{5}=\ln\;(1.2)-\ln\;(0.8)=0.405$ .

**Heterogeneous treatment effect modification**: In addition to a common treatment effect modification as outlined above, we considered a heterogeneous (random) treatment effect modification scenario. A distinctive feature of this scenario is that there is a distribution, as opposed to fixed values, of regression coefficients that govern the magnitude and direction of the effect modification underlying all trials included in the IPD meta-analysis. The set of regression coefficients underlying the data for the j th study, compactly denoted as $\beta_{j}^{*}=(\beta_{1}^{*},\ldots ,\beta_{5}^{*})$ , followed a multivariate normal distribution centred around the average of β and an identity variance/covariance matrix with a common variability across studies $\sqrt{\tau^{2}}\cdot I_{5}$ . The regression coefficients underlying the j th study denoted by $\beta_{j}^{*}$ were randomly picked from $\beta_{j}^{*}∼MVN(\beta ,\sqrt{\tau^{2}}\cdot I_{5})$ , where the between-study variability was set to τ=0.05 for all coefficients. To facilitate a comparison with the common effect scenario, the vector β was defined with the identical average parameter values of β=(−0.223,0.182,0.405,0.223,0.405) .

### 3.2. Scenarios

We generated IPD meta-analyses under a common and heterogeneous treatment effect modification with N=6 and N=12 number of trials. The proportion of trials with systematic missing EM was set to 1/6 and 1/3 following the approach presented in Jolani et al.,^10^ Audigier et al.^14^ The EM was set to missing at a single site independently of all observed covariates. We can reasonably assume that data on the EM might be missing due to administrative, financial, and/or study design reasons that are unrelated to other observed information but related to the study site itself. For all scenarios, the sample size for the studies with complete information was fixed at n=500 participants (with an average of 127 events per study). In total, we simulated eight main scenarios each with two different imputation models according to the number of product terms between the treatment and survival time/censoring indicator included in the imputation model. Each scenario was simulated 1000 times with each 30 imputations. As a reference scenario we considered a complete case analysis.

### 3.3. Imputation models

In all scenarios, we imputed the systematically missing values for z with two different specifications of the imputation model in the same IPD meta-analysis. Given the operating mechanism between treatment and EM in predicting mortality, a congenial imputation model for z should include main effects and the product terms between the treatment and mortality (both survival time and censoring indicator) in predicting z . We used the Nelson–Aalen estimator of the cumulative hazard rate, $\hat{H}(t)$ , in each study as explained by White et al.^26^ The imputation model was specified according to equation 1 in the description of CQI with the probability that $\theta_{ik}$ is equal to level k of the EM conditionally on the set of observed variables $w_{i}$ . Here, $w_{i}$ is a vector of variables with $w_{i}=(x_{i},{\hat{H}}_{i}(t),d_{i})$ . The imputation model was fit in studies with complete data on the EM. The first specification of the imputation model with all product terms (Model I: two product terms) imputing the systematic missing variable was defined as:

(10) $$\ln\;\left(\frac{\theta_{ik}}{\theta_{i0}}\;|\;x_{i},{\hat{H}(t)}_{i},d_{i}\right)=\gamma_{0k}+\gamma_{1k}x_{i}+\gamma_{2k}{\hat{H}(t)}_{i}+\gamma_{3k}d_{i}+\gamma_{4k}x_{i}\cdot {\hat{H}(t)}_{i}+\gamma_{5k}x_{i}\cdot d_{i}$$

The imputation model is congenial with the substantive outcome model and should be derived after careful consideration as outlined in White et al.^17^

The following imputation model differs from the initial one in a way that it is incomplete. We specified the imputation model omitting all product terms, $x_{i}\cdot {\hat{H}(t)}_{i}$ and $x_{i}\cdot d_{i}$ (Model II: zero product terms).

(11) $$\ln\;\left(\frac{\theta_{ik}}{\theta_{i0}}\;|\;x_{i},{\hat{H}(t)}_{i},d_{i}\right)=\gamma_{0k}+\gamma_{1k}x_{i}+\gamma_{2k}{\hat{H}(t)}_{i}+\gamma_{3k}d_{i}$$

### 3.4. Estimation and statistical inference

Based on the observed and imputed data of the trials included in the IPD meta-analysis, a two-stage approach was used to obtain an estimate of the regression coefficients of the multivariable Weibull survival model. In a two-stage approach, study-specific regression coefficients are first estimated within each study based on imputed data and then combined across studies with a multivariate meta-regression model according to a common or heterogeneous treatment effect modification.^27^ The inverse variance method and the restricted maximum likelihood were used for the common and heterogeneous (random) treatment effect modification, respectively.^24^ Rubin’s rules were used to aggregate the estimates of the regression coefficients across imputations. Following standard notations,^25^ we briefly outline Rubin’s rules for a single parameter. Starting from the M different ${\hat{\beta }}_{m}$ , the MI point estimate is their average: ${\hat{\beta }}_{MI}=1/M\sum_{m=1}^{M}{\hat{\beta }}_{m}$ . The variance of ${\hat{\beta }}_{MI}$ , $\sigma_{MI}$ , is estimated by the sum of a within imputation variance, $W_{MI}$ , and a between imputation variance, $B_{MI}$ . A (1+1/M) term is added to account for the finite number of imputations:

(12) $$\begin{array}{rl} W_{MI} & =\frac{1}{M}\sum_{m=1}^{M}{\hat{\sigma }}_{m}^{2}\\ B_{MI} & =\frac{1}{M-1}\sum_{m=1}^{M}({\hat{\beta }}_{k}-\beta_{MI}{)}^{2}\\ \sigma_{MI} & =W_{MI}+\left(1+\frac{1}{M}\right)B_{MI} \end{array}$$

Furthermore, a multivariate Wald type test, conducted with a Type I error of 5%, for the hypothesis of no effect modification translated into testing that the two regression coefficients $\beta_{4}$ and $\beta_{5}$ of the treatment EM product terms, were jointly equal to zero.

### 3.5. Performance measures

The principle measures to assess the performance of the two-stage imputation method were bias and coverage.^20^ Bias was computed as the distance between the average estimated regression coefficients, ( $\beta_{1},\beta_{4},\beta_{5},\beta_{1}+\beta_{4},\beta_{1}+\beta_{5}$ ), and the set parameters ( $\beta_{1}=-0.223,\beta_{4}=0.223,\beta_{5}=0.405,\beta_{1}+\beta_{4}=0.000,\beta_{1}+\beta_{5}=0.182$ ). Coverage was computed as the fraction of studies in which the estimated 0.025 and 0.975 quantiles of confidence covered the parameter values. In addition, empirical standard error (ESE) and mean squared error (MSE) were reported.

### 3.6. Results

**Figure 1.**
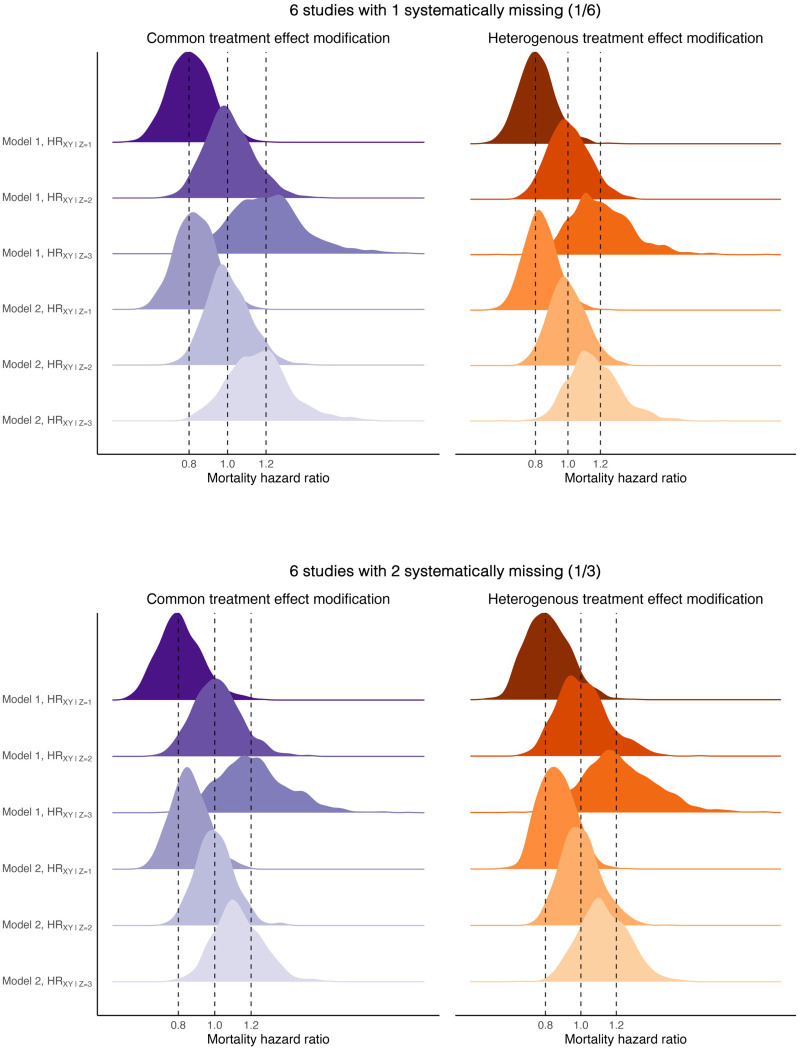
Sampling distribution of the estimated mortality hazard ratios conferred by the treatment at different levels of the low ( z=1 ), null ( z=2 ), and high ( z=3 ) effect modifiers (EMs) under different complexities of the imputation model for common and heterogeneous treatment effect modification. Both scenarios, common and heterogeneous treatment effect modification, were complied of 6 studies with 1/6 and 1/3 of the studies with systematically missing information on the EM. The sample size of all studies was set to 500. Model 1 (two product terms): $x_{i}\cdot \hat{H}(t{)}_{i}$ and $x\cdot d_{i}$ . Model 2 (zero product terms): Omitting $x\cdot \hat{H}(t)$ and $x\cdot d_{i}$ .

**Figure 2.**
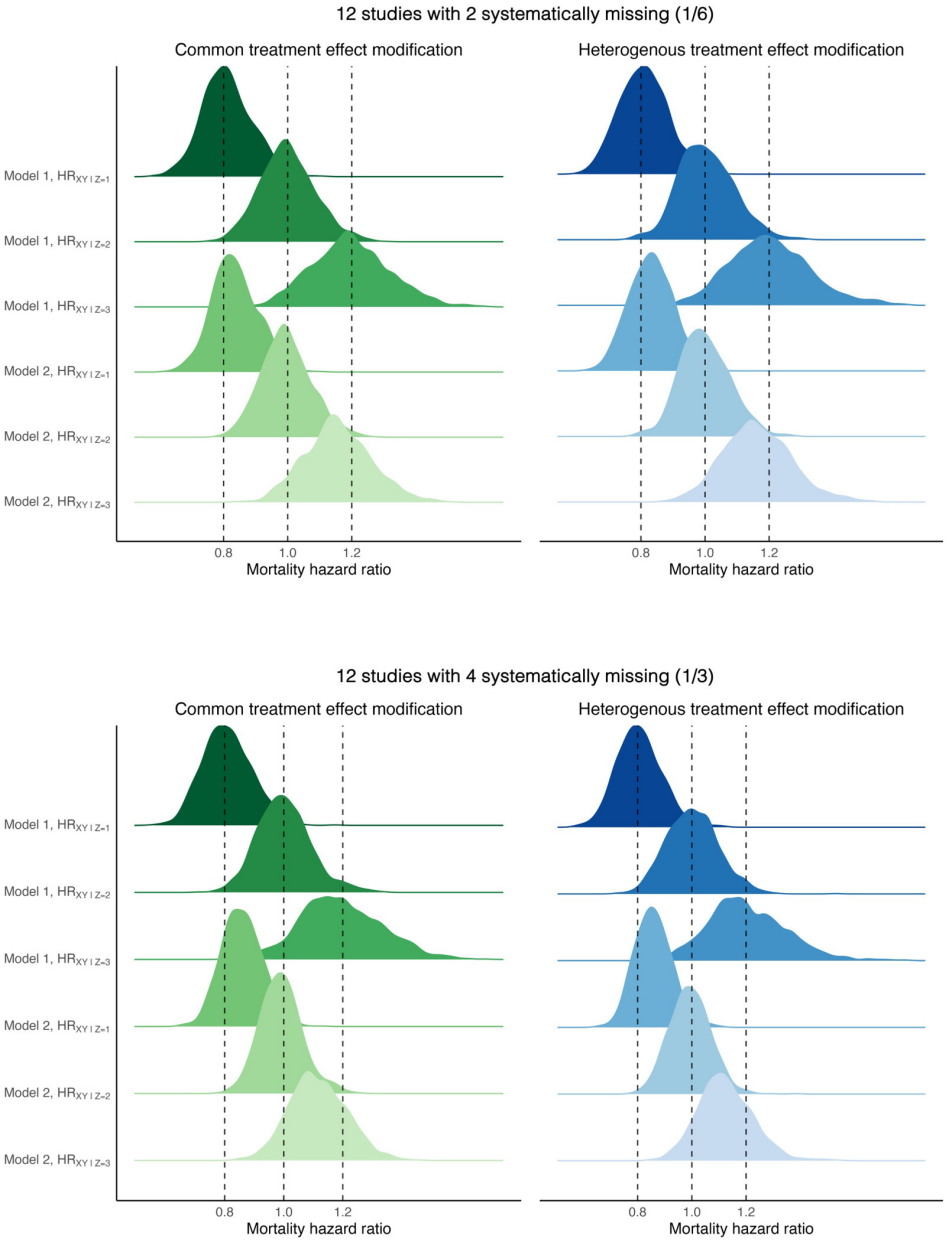
Sampling distribution of the estimated mortality hazard ratios conferred by the treatment at different levels of the low ( z=1 ), null ( z=2 ), and high ( z=3 ) effect modifiers(EMs) under different complexities of the imputation model for common and heterogeneous treatment effect modification. Both scenarios, common and heterogeneous treatment effect modification, were complied of 12 studies with 1/6 and 1/3 of the studies with systematically missing information on the EM. The sample size of all studies was set to 500. Model 1 (two product terms): $x_{i}\cdot \hat{H}(t{)}_{i}$ and $x\cdot d_{i}$ . Model 2 (zero product terms): Omitting $x\cdot \hat{H}(t)$ and $x\cdot d_{i}$ .

The results of the performance of CQI in all scenarios for the common and heterogeneous treatment effect modification are shown in Tables 1 and 2, respectively. The average estimates of the regression coefficients describing the effect of the treatment, x , at different levels of the EM, z , are presented in addition to bias and coverage. Figures 1 and 2 show the different distributions of the parameters under the two specifications of the imputation model with 6 and 12 studies, respectively. Bias was comparably low for both common and heterogeneous treatment effect modification and no stark differences could be pointed out between the number of studies with systematically missing data in the IPD meta-analysis. The ESEs were lower in scenarios with 12 studies whereas more uncertainty was observed in smaller IPD meta-analyses. The complete case analysis was unbiased and coverage of the effect estimates was close to 95%. The following two sections describe in more detail the results for the common and heterogeneous treatment effect modification.

**Table 1.** Log hazard ratios (average and performance measure) for the treatment effect at different levels of the EM by the complexity of the imputation models and by the size of the studies with systematic missing data on the EM.

	Complete case analysis					Model I: Two product terms					Model II: Zero product terms				
Parameters	${\hat{\beta }}_{1}$	${\hat{\beta }}_{4}$	${\hat{\beta }}_{5}$	${\hat{\beta }}_{1}+{\hat{\beta }}_{4}$	${\hat{\beta }}_{1}+{\hat{\beta }}_{5}$	${\hat{\beta }}_{1}$	${\hat{\beta }}_{4}$	${\hat{\beta }}_{5}$	${\hat{\beta }}_{1}+{\hat{\beta }}_{4}$	${\hat{\beta }}_{1}+{\hat{\beta }}_{5}$	${\hat{\beta }}_{1}$	${\hat{\beta }}_{4}$	${\hat{\beta }}_{5}$	${\hat{\beta }}_{1}+{\hat{\beta }}_{4}$	${\hat{\beta }}_{1}+{\hat{\beta }}_{5}$
6 studies with 1 systematically missing (1/6 missing)															
Average	−0.217	0.221	0.396	0.003	0.178	−0.217	0.219	0.394	0.002	0.176	−0.186	0.184	0.330	−0.002	0.143
Bias	0.006	−0.002	−0.010	0.003	−0.004	0.006	−0.004	−0.012	0.002	−0.006	0.037	−0.039	−0.076	−0.002	−0.039
Coverage^1^	94.7	95.3	95.0	94.7	93.6	94.5	95.1	94.9	94.9	93.6	96.7	97.8	96.8	96.8	95.7
ESE	0.143	0.188	0.210	0.127	0.158	0.139	0.187	0.209	0.121	0.154	0.124	0.157	0.175	0.110	0.135
MSE	0.021	0.035	0.044	0.016	0.025	0.019	0.035	0.044	0.015	0.024	0.017	0.026	0.036	0.012	0.020
6 studies with 2 systematically missing (1/3 missing)															
Average	−0.225	0.230	0.408	0.005	0.184	−0.221	0.227	0.402	0.006	0.181	−0.157	0.153	0.272	−0.004	0.115
Bias	−0.001	0.006	0.003	0.005	0.001	0.002	0.004	−0.003	0.006	−0.001	0.066	−0.070	−0.134	−0.004	−0.068
Coverage^2^	95.1	95.6	95.2	94.9	95.3	93.9	94.4	94.8	93.8	95.5	96.3	99.0	97.1	98.2	97.5
ESE	0.158	0.212	0.231	0.140	0.168	0.149	0.209	0.228	0.128	0.157	0.115	0.142	0.154	0.103	0.116
MSE	0.025	0.045	0.053	0.020	0.028	0.022	0.044	0.052	0.016	0.024	0.018	0.025	0.042	0.011	0.018
12 studies with 2 systematically missing (1/6 missing)															
Average	−0.216	0.214	0.393	−0.002	0.176	−0.215	0.212	0.391	−0.003	0.175	−0.185	0.178	0.327	−0.007	0.142
Bias	0.007	−0.009	−0.013	0.002	−0.006	0.008	−0.011	−0.015	−0.003	−0.007	0.038	−0.045	−0.078	−0.007	−0.040
Coverage^3^	96.5	94.6	96.1	94.2	96.5	95.7	94.0	95.9	93.7	95.7	97.0	96.2	96.7	96.3	95.8
ESE	0.097	0.130	0.138	0.089	0.104	0.094	0.130	0.137	0.085	0.102	0.084	0.109	0.115	0.077	0.090
MSE	0.009	0.017	0.019	0.008	0.011	0.009	0.017	0.019	0.007	0.010	0.008	0.014	0.019	0.006	0.010
12 studies with 4 systematically missing (1/3 missing)															
Average	−0.220	0.218	0.394	−0.003	0.173	−0.217	0.215	0.389	−0.002	0.172	−0.156	0.145	0.262	−0.011	0.106
Bias	0.003	−0.006	−0.012	−0.003	−0.009	0.006	−0.008	−0.016	−0.002	−0.011	0.067	−0.078	−0.143	−0.011	−0.076
Coverage^4^	95.4	95.2	95.8	95.2	95.4	95.4	94.5	95.1	93.6	94.0	95.0	97.9	94.0	97.8	95.2
ESE	0.110	0.146	0.162	0.098	0.123	0.102	0.144	0.158	0.089	0.115	0.079	0.097	0.107	0.071	0.086
MSE	0.012	0.021	0.026	0.010	0.015	0.010	0.021	0.025	0.008	0.013	0.011	0.016	0.032	0.005	0.013

Results are presented for 1/6 and 1/3 of the studies with systematically missing data. All simulated data was generated under a **common** treatment effect modification with parameter values equal to: $\beta_{1}=-0.223,\beta_{4}=0.223,\beta_{5}=0.405,\beta_{1}+\beta_{4}=0.000,\beta_{1}+\beta_{5}=0.182$ . Each simulated IPD meta-analysis was analysed with a two-stage multivariate Weibull survival model. Each scenario was simulated 1000 times with each with 30 imputations using conditional quantile imputation, ESE, MSE. **Model I (two product terms)**: $x_{i}\cdot \hat{H}(t{)}_{i}$ and $x\cdot d_{i}$ . **Model II (zero product terms)**: Omitting $x\cdot \hat{H}(t)$ and $x\cdot d_{i}$ . MCE was below a maximum of 0.007 for all parameters except coverage; ^1^:MCE < 0.891; ^2^:MCE < 1.042; ^3^:MCE < 1.024; ^4^:MCE < 1.550. IPD: individual participant data; EM: effect modifier; ESE: empirical standard error; MSE: mean squared error; MCE: Monte Carlo error.

**Table 2.** Log hazard ratios (average and performance measure) for the treatment effect at different levels of the EM by the complexity of the imputation models and by the size of the studies with systematically missing data on the EM.

	Complete case analysis					Model I: Two product terms					Model II: Zero product terms				
Parameters	${\hat{\beta }}_{1}$	${\hat{\beta }}_{4}$	${\hat{\beta }}_{5}$	${\hat{\beta }}_{1}+{\hat{\beta }}_{4}$	${\hat{\beta }}_{1}+{\hat{\beta }}_{5}$	${\hat{\beta }}_{1}$	${\hat{\beta }}_{4}$	${\hat{\beta }}_{5}$	${\hat{\beta }}_{1}+{\hat{\beta }}_{4}$	${\hat{\beta }}_{1}+{\hat{\beta }}_{5}$	${\hat{\beta }}_{1}$	${\hat{\beta }}_{4}$	${\hat{\beta }}_{5}$	${\hat{\beta }}_{1}+{\hat{\beta }}_{4}$	${\hat{\beta }}_{1}+{\hat{\beta }}_{5}$
6 studies with 1 systematically missing (1/6 missing)															
Average	−0.221	0.220	0.399	−0.001	0.179	−0.221	0.219	0.397	−0.002	0.176	−0.189	0.183	0.333	−0.007	0.143
Bias	0.003	−0.003	−0.006	−0.001	−0.003	0.002	−0.004	−0.009	−0.002	−0.006	0.034	−0.041	−0.073	−0.007	−0.039
Coverage^1^	94.8	95.6	94.9	94.1	94.9	94.6	95.3	94.7	93.7	95.2	96.5	98.0	96.7	96.2	97.3
ESE	0.143	0.186	0.208	0.130	0.159	0.138	0.186	0.207	0.125	0.153	0.123	0.155	0.174	0.113	0.135
MSE	0.020	0.035	0.043	0.017	0.025	0.019	0.034	0.043	0.016	0.023	0.016	0.026	0.035	0.013	0.020
6 studies with 2 systematically missing (1/3 missing)															
Average	−0.219	0.222	0.405	0.003	0.186	−0.217	0.219	0.400	0.002	0.183	−0.152	0.146	0.267	−0.007	0.114
Bias	0.004	−0.001	−0.000	0.003	0.004	0.006	−0.004	−0.006	0.002	0.000	0.070	−0.077	−0.138	−0.007	−0.068
Coverage^2^	94.9	94.3	94.8	93.1	95.6	94.9	93.0	92.5	91.5	94.5	97.5	98.7	96.9	97.6	97.7
ESE	0.162	0.220	0.237	0.150	0.174	0.150	0.219	0.235	0.140	0.166	0.116	0.147	0.158	0.111	0.122
MSE	0.026	0.048	0.056	0.022	0.030	0.023	0.048	0.055	0.020	0.028	0.018	0.028	0.044	0.012	0.019
12 studies with 2 systematically missing (1/6 missing)															
Average	−0.219	0.220	0.400	0.001	0.181	−0.218	0.219	0.398	0.001	0.180	−0.187	0.184	0.334	−0.003	0.147
Bias	0.004	−0.003	−0.005	0.001	−0.001	0.005	−0.004	−0.008	0.001	−0.002	0.036	−0.040	−0.072	−0.003	−0.035
Coverage^3^	96.6	96.2	96.4	95.2	95.4	96.2	96.6	95.9	95.0	94.6	96.6	97.8	96.5	96.8	96.4
ESE	0.093	0.126	0.019	0.088	0.107	0.091	0.126	0.138	0.085	0.105	0.082	0.106	0.116	0.077	0.092
MSE	0.009	0.016	0.140	0.008	0.011	0.008	0.016	0.019	0.007	0.011	0.008	0.013	0.019	0.006	0.010
12 studies with 4 systematically missing (1/3 missing)															
Average	−0.225	0.223	0.407	−0.002	0.181	−0.220	0.220	0.401	−0.000	0.180	−0.158	0.148	0.271	−0.009	0.113
Bias	−0.002	−0.000	0.001	−0.002	−0.001	0.002	−0.003	−0.004	−0.000	−0.002	0.065	−0.075	−0.135	−0.009	−0.069
Coverage^4^	95.6	94.9	96.0	94.3	94.9	94.5	93.4	94.9	93.1	93.2	95.9	97.6	93.8	97.7	95.7
ESE	0.111	0.152	0.166	0.103	0.126	0.102	0.151	0.164	0.095	0.119	0.078	0.102	0.111	0.076	0.089
MSE	0.012	0.023	0.028	0.011	0.016	0.010	0.023	0.027	0.009	0.014	0.010	0.016	0.030	0.006	0.013

Results are presented for 1/6 and 1/3 of the studies with systematically missing data. All simulated data was generated under a **heterogeneous** treatment effect modification with the average parameter values equal to: $\beta_{1}=-0.223,\beta_{4}=0.223,\beta_{5}=0.405\beta_{1}+\beta_{4}=0.000,\beta_{1}+\beta_{5}=0.182$ . Each simulated IPD meta-analysis was analysed with a two-stage multivariate Weibull survival model. Each scenario was simulated 1000 times with each with 30 imputations using conditional quantile imputation, ESE, MSE. **Model I (two product terms)**: $x_{i}\cdot \hat{H}(t{)}_{i}$ and $x\cdot d_{i}$ . **Model II (zero product terms)**: Omitting $x\cdot \hat{H}(t)$ and $x\cdot d_{i}$ . MCE was below a maximum of 0.007 for all parameters except coverage; ^1^:MCE < 0.828; ^2^:MCE < 1.063; ^3^:MCE < 1.024; ^4^:MCE < 1.556. IPD: individual participant data; EM: effect modifier; ESE: empirical standard error; MSE: mean squared error.

#### 3.6.1. Common treatment effect modification

The absolute value of the bias after using CQI to impute the systematically missing values for the EM was less than 0.016 for all effect estimates for the specification of the imputation model with two product terms. Bias was comparably low for scenarios with 6 and 12 studies, regardless of the number of studies with systematically missing data on the EM. The largest bias with the congenial imputation model across all scenarios was found for the scenario with 12 studies when 1/3 of the studies had systematically missing data on the EM with 0.006, −0.002 , and −0.011 for the beneficial, null, and harmful treatment effect of the EM, respectively. Specifying the imputation with zero product terms led to larger biases in all effect estimates throughout the different scenarios. Here, the largest bias was found for 12 studies when 1/3 of the studies had systematically missing values on the EM, at around 0.067, −0.011 , and −0.076 for the beneficial, null, and harmful treatment effect of the EM, respectively. Figures 1 and 2 show the difference in sampling distribution of the different levels of the EM between the two specifications of the imputation model. The ability of the IPD meta-analysis to differentiate between levels of the EM decreased drastically when the imputation model included only main effects of treatment, time, and censoring indicator. Here, the magnitude of the effect modification was close to 0, making it difficult for the statistical test to reject the null hypothesis of no effect modification.

The coverage of the treatment effect estimates were close to 95% in all scenarios. Coverage was at 95.7%, 93.7%, and 95.7% for the beneficial, null, and harmful treatment effect of the EM, respectively, for 12 studies when a sixth of studies had systematic missing values on the EM. With a third of the studies having systematically missing data, the coverage for the same scenario remained very similar at 95.4%, 93.6%, and 94.0% for the beneficial, null, and harmful treatment effect of the EM, respectively. The small differences in coverage are explained by random variability in the estimates. When the imputation model included no product terms, coverage was 95.0%, 97.8%, and 95.2% for the three levels of the EM, respectively.

Notably, the precision in all effect estimates increased after using MI to retain trials with systematically missing values on the EM. The largest gains in precision were made when a higher proportion of studies with missing data was imputed. This is evident in the consistently smaller ESE and corresponding MSE between the complete case analysis and MI with the congenial specification of the imputation model. For example, the ESE for the complete case analysis in the scenario of 12 trials with 4 trials having systematically missing data was 0.110, 0.098, and 0.123 for the low, null, and harmful treatment effect, respectively. After retaining the four trials in the analysis, the ESE was reduced to 0.102, 0.089, and 0.115, respectively. In addition, the MSE was lower (about 50%) for the scenarios with 12 studies compared to the scenario with only six studies, regardless of the number of studies with systematically missing data on the EM. Table 1 contains all values for the scenarios of the common treatment effect modification.

#### 3.6.2. Heterogeneous treatment effect modification

Overall, bias was lower compared to the scenarios of common treatment effect modification. In all scenarios simulated under a heterogeneous treatment EM, the absolute value of the bias remained less than 0.009 for all effect estimates under the congenial specification of the imputation model. There were no stark differences in the magnitude of bias between the scenarios. With 12 studies, when 1/3 of the studies were imputed, the bias for all levels of the EM remained below 0.002 under the specification of the imputation model with two product terms. Under the incomplete (zero product terms) specification of the imputation model, large bias was introduced in all parameters in all scenarios. The absolute value of the bias increased up to 0.069 for the estimates of the EM. Here, with 1/3 of the studies having systematically missing data on the EM, bias was highest for the additional effect of the harmful EM ( $\beta_{5}$ ) with −0.138 and −0.135 for 6 and 12 studies, respectively.

Coverage remained close to 95% in most scenarios of the heterogeneous treatment effect modification. For 6 studies with 1/3 of the studies being imputed, the coverage was at 94.9%, 91.5%, and 94.5% for the beneficial, null, and harmful treatment effect of the EM. For the same scenario with 12 studies, coverage levels were at 94.5%, 93.1%, and 93.2%, respectively. Here, the coverage of the interaction terms itself was marginally closer to the nominal level at 94.5% and 95.1%.

Similar to the common treatment effect modification, precision improved when using MI to retain trials with systematically missing values on the EM, most notable in the smaller ESE and MSE values compared to the complete case analysis. Additionally, the MSE was 50% lower in the scenarios with 12 studies, compared to those with 6.

In summary, in all scenarios under the specification of the congenial imputation model, no substantial bias was found in estimates at all levels of the EM. The magnitude of the bias was not affected with an increase in the number of studies with systematic missing values on the EM. The specification of the imputation model with zero product terms always resulted in an increase in bias of the estimates, regardless of the number of studies with systematically missing data on the EM. Using a two-stage imputation approach to impute systematically missing EM values, we observed an improvement in the precision of effect estimates in all scenarios under the congenial specification of the imputation model compared to the complete case analysis.

## 4. The effect of PORT on survival at different stages of the disease

In this section, we illustrate the use of CQI for a systematically missing EM in an IPD meta-analysis of trials. The main question was about the effectiveness of PORT in patients with completely resected non-small cell lung cancer at different stages of the disease. A multivariate IPD meta-analysis of PORT versus surgery alone on survival in patients with resected non-small-cell lung cancer at different stages of the disease was performed.^28^ The outcome in all trials was time from randomisation to death from all causes or censoring, whichever came first. The disease stage of the patients was measured in three different levels ranging from stage I to III. The 10 trials included in this study had a varying sample size ranging from 69 to 316 participants per trial with a total of 1642 participants and 1082 deaths across all 10 trials. A common treatment effect modification underlying the trials was estimated with a two-stage multivariable Cox proportional hazard regression model with x being the treatment and z the EM (disease stage):

(13) $$h(t|x_{i},z_{i})=h_{0}(t)\cdot e^{\beta_{1}x_{i}}+\beta_{2}I(z_{i}=2)+\beta_{3}I(z_{i}=3)+\beta_{4}x_{i}\cdot I(z_{i}=2)+\beta_{5}x_{i}\cdot I(z_{i}=3)$$

where $h_{0}(t)$ is the baseline hazard function. The function $h_{0}(t)$ indicates some unspecified trend of the hazard over time when all predictors are zero. Seven out of the 10 trials had complete information on the disease stage of patients (the EM). However, three trials with a sample size of 110, 169, and 176 had no information on the disease stage, i.e., systematically missing information. Different from the settings in the simulation study, age and sex of the patients were measured and could also be used for the imputation model. CQI was used to impute the systematically missing data on the disease stage in three trials based on the trials with complete information on the stage of the disease of patients (30 imputations). This allowed us to retain all 10 trials in the analysis and not lose valuable treatment-outcome information. In the seven studies with complete data on disease stage, the imputation model was specified in accordance to the final outcome model, incorporating the effect modification between the time-varying and indicator variable for death:

(14) $$\begin{array}{rl} \ln\;\left(\frac{\theta_{ik}}{\theta_{i0}}\;|\;x_{i},{\hat{H}(t)}_{i},d_{i},age_{i},sex_{i}\right) & =\gamma_{0k}+\gamma_{1k}x_{i}+\gamma_{2k}{\hat{H}(t)}_{i}+\gamma_{3k}d_{i}\\ & \;+\gamma_{4k}x_{i}\cdot {\hat{H}(t)}_{i}+\gamma_{5k}x_{i}\cdot d_{i}+\gamma_{6k}age_{i}+\gamma_{7k}sex_{i} \end{array}$$

The data used in this paper is a publicly available, real-data based simulated example with similar characteristics to the PORT data and is only used for the illustration of the presented imputation method.^29^

### 4.1. Results

In this sample of data, only using the seven studies with complete information on disease stage, the statistical test did not provide a strong indication against the hypothesis of a homogeneous effect of PORT on mortality across stages of the disease ( $\chi^{2}=5.00$ , p=0.082 ). The estimated mortality HRs of PORT were 1.00, 1.02, and 1.43, for the first, second, and third stage of the disease, respectively. At the latest stage of the disease, the effect of PORT on survival was estimated with a 43% increase in the mortality hazard rate (HR = 1.43, 95% CI: 1.11, 1.85, z=2.73 , p=0.006 ).

Similar to the complete case analysis, the statistical test when using all 10 studies after using CQI to impute the systematically missing values for disease stage in three trials did not provide a clear indication against the hypothesis of a homogeneous effect of PORT on mortality across stages of the disease ( $\chi^{2}=5.53$ , p=0.063 ). For the first, second, and third stage of the disease the estimated mortality HRs of PORT were 1.03, 1.03, and 1.45, respectively. The mortality HR increased by 45% ( z=3.14 , p=0.002 ) when using PORT compared to surgery alone at disease stage III (HR = 1.45, 95% CI: 1.15, 1.83) (see Table 3).

**Table 3.** Mortality HRs, 95% CIs, and SEs conferred by postoperative radiotherapy at different stages of the disease in complete case and MI datasets.

	7 studies (Complete case)			10 studies (Using MI)		
	N = 1187, deaths = 785			N = 1642, deaths = 1082		
Mortality HR (95% CI)	HR	95% CI	SE	HR	95% CI	SE
Stage I	1.00	(0.78, 1.27)	0.13	1.03	(0.82, 1.28)	0.12
Stage II	1.02	(0.80, 1.29)	0.12	1.03	(0.83, 1.28)	0.11
Stage III	1.43	(1.11, 1.85)	0.19	1.45	(1.15, 1.83)	0.17

The individual participant data meta-analysis included 10 trials, 7 with complete information on the disease stage, 3 with systematically missing data on disease stage. Conditional quantile imputation was used to impute the systematically missing data based on the trials with complete information. Data were analysed with a two-stage multivariable Cox-regression model. MI: multiple imputation; HR: hazard ratio; CI: confidence interval; SE: standard error.

### 4.2. Interpretation

The described example of imputing the three trials with systemically missing values on disease stage suggests a beneficial use of MI in this scenario. While the empirical results of the analysis did not change substantially compared to the complete case analysis, all studies are retained in the analysis without introducing substantial bias and pointing towards a harmful effect of PORT on survival in the later stage of the disease. By using MI to retain the three trials with systematically missing values on disease stage, we were able to include an additional 455 participants including 297 mortality cases in the analysis. While no substantial differences in the mortality HRs between the complete case analysis and the analysis with MI were estimated, the precision in the effect estimates at all levels of disease stage increased, without distorting the association that was estimated in the complete case analysis. This is in line with the results presented from the simulation study. Overall, in the example of the effect of PORT on survival at different stages of the disease, using MI to retain three trials with systematically missing values on disease stage indicates a worthwhile use to increase the generalisbility and precision of clinical effect measures without introducing bias.

## 5. Discussion

This simulation study evaluated a two-stage imputation method based on conditional quantiles to assess its performance on retaining studies with systematically missing EMs in IPD meta-analyses. We evaluated the feasibility of imputing systematically missing EM in IPD meta-analysis with a limited number of trials (6 and 12) under a common and heterogeneous treatment effect modification. The results demonstrated that the bias for all levels of the EM was considerably low for the common and heterogeneous treatment effect modification. Compared to the complete case analysis, using the two-stage imputation approach to retain all trials with missing data in the analysis improved the precision of effect estimates in all scenarios under a congenial specification of the imputation model. The bias increased for all scenarios when the imputation model was incompletely specified, i.e., missing important product terms.

### 5.1. Performance of CQI

First, the proposed approach, CQI, indicated no substantial difference in bias between smaller and larger IPD meta-analysis nor substantial differences when the number of studies with systematically missing data on the EM doubled (i.e., from one sixths to one third). In fact, the performance showed a negligible bias for both common and heterogeneous treatment effect modification IPD meta-analysis that is comparable with biases reported for other MI methods.^14^ The average effect estimates after using CQI were comparable with those of the complete case analysis. Even though a complete case analysis can be justified when assuming missing completely at random (MCAR), it is suboptimal when the fraction of studies with missing EM is large. As we demonstrated in this simulation study, this can lead to a reduction in the precision of effect estimates and substantial loss of data. Bias increased substantially when the imputation model was incompletely specified with zero product terms (i.e., not congenial with the substantive outcome model). This is in line with previous work that has demonstrated the importance of correctly specifying the imputation model, whereby failing to do so leads to biased inference.^16^ Sensitivity analyses are often used in practice to explore the implications of different imputation models.^30–32^

Second, coverage was close to the nominal level for all scenarios that were assessed. Some small deviations from the desired 95% were observed in scenarios with a larger number of studies and when a third of the studies had systematically missing data on the EM. In the same scenarios, similar deviations from 95% were seen for the complete case analysis. This is in line with simulation results presented by Resche-Rigon and White^11^ indicating slight undercoverage in the presence of only systematically missing data.

Third, we showed that using a two-stage imputation approach in the context of systematically missing data in IPD meta-analysis improved the precision of effect estimates compared to the complete case analysis. That is, lower ESE and MSE across all scenarios were estimated reflecting the better use made of the data. In addition, by including all trials in the analysis, the generalisbility of study findings potentially increases, although it is difficult to quantify this numerically. In summary, CQI resulted in an analysis with (1) low bias, (2) coverage close to the nominal 95% level, and (3) small variance.

### 5.2. Assumptions

Despite satisfactory performance benefits of the proposed method, certain assumptions need to be discussed. First, we assumed that the underlying treatment effect at different levels of the EM is consistent across all trials. This assumption supports the interpretation of observed variations in treatment effects across levels of the EM as true interactions, as opposed to artefacts from trial-level heterogeneity, such as differences in study design, populations, or implementation. Second, we assumed that all trials collected similar information on other covariates used to inform the imputation model. This assumption is more likely to be satisfied in prospective IPD meta-analyses, where common data collection, analysis plans, and harmonisation strategies are implemented, thereby reducing the risk of systematic missing values.^33^ In retrospective IPD meta-analyses, however, data collection may have occurred at different time points and protocols may vary across studies, increasing the risk of systematic missingness.^34^ Related to this, we also assumed that the EM has the same number of categories (or levels) across trials and was measured consistently across studies. In IPD meta-analyses, harmonisation of covariates is a crucial step prior to implementing any MI approach. Therefore, EMs with different distributions across trials must be harmonised before applying the two-stage imputation approach. Last, we addressed systematic missing data under a MCAR assumption. As previously mentioned, a complete case analysis is theoretically justifiable, albeit suboptimal due to the significant loss of data. Under a MAR assumption, the approach to impute systematically missing values of the EM would not differ. While the results may become more sensitive to the choice of predictors included in the imputation model assuming MAR, simulation studies have shown small performance differences between MCAR and MAR in multilevel data settings for sporadically and systematically missing data.^11,35^

### 5.3. Limitations

Despite demonstrating an approach for imputing a systematically missing EM in IPD meta-analyses with a limited number of trials, this study is subject to a number of limitations. First, we aimed at simulating simplified, yet complex enough, scenarios that are related to realistic challenges in IPD meta-analysis, whilst remaining accessible for researchers that are faced with such problems. While there is an abundance of scenarios that were not considered, we are confident that the chosen scenarios can give an intuition about the performance of the CQI method and observation of a general trend of when MI is worth considering in IPD meta-analysis to impute systematically missing EM. Second, we solely focussed on the impact of systematically missing data and did not consider scenarios with sporadically and systematically missing data at the same time. Third, in all our simulations we used 30 imputations. We did not test whether the performance, in particular the coverage, changes with an increasing number of imputations due to reasonable computation time. Last, we did not consider larger degrees of heterogeneity across studies in our simulations due to the focus on IPD meta-analyses with a limited number of studies.

### 5.4. Phases of methodological development

Based on the phases of methodological research in biostatistics according to Heinze et al.,^36^ this work can be categorised as part of phase I to II developments of a two-stage imputation procedure for systematic missing covariates in IPD meta-analysis in line with previous work.^11^ Facilitating software is available in Stata and provided in the resources linked to this study. Further analysis is needed to test the approach in a variety of settings such as different outcomes, varying degrees of heterogeneity and DGMs. In particular, future continuation on the refinement of this approach can be directed towards:

(1) Extending the approach to multivariate imputation as proposed in Resche-Rigon and White:^11^ Further software implementation of CQI into MI with chained equation is a logical step to make it more widely applicable in scenarios with sporadically and systematically missing values. An extension of CQI to continuous systematic missing covariates using quantile regression has been recently presented.^37^
(2) Integrating random effects into the two-stage imputation procedure to increase heterogeneity between imputed datasets: Given the small number of studies in this analysis, a random-effects model would provide a poor estimate of the variability between studies. However, using a common-effects model to derive average regression coefficients for assigning values to the missing EM can result in more homogeneous imputed datasets.

### 5.5. Conclusion

This simulation study presented and evaluated the use of a two-stage imputation procedure - CQI - to impute systematically missing EMs in IPD meta-analyses with a limited number of trials. The absolute bias for common and heterogeneous effect IPD meta-analyses was less than 0.016 and 0.007, respectively, with coverage close to its nominal value across all levels of the EM. In addition, CQI improved the precision of pooled effect estimates compared to a complete case analysis excluding trials with systematically missing values on the EM. An incomplete specification of the imputation model resulted in biased inference even if the proportion of studies with systematically missing data was small.
